# Jimmies

**Published:** 1885-07

**Authors:** 


### Jimmies.

These formidable appliances are made, it appears, in regular gradations of size, the three largest being known as the "Lord Mayor," the "Alderman," and the "Common Councilman." The Lord Mayor is 4 feet 3 inches in length, and is only used on great occasions, say the breaking open of a strong room or very heavy safe. The Alderman is 3 feet 3 inches in length ; the Common Councilman about 2 inches shorter, and, as befits its lower dignity, not quite so stout. Whatever may be said as to the projected reform of the city of London, our readers will agree with us that the sooner this corporation is abolished the better. Passing downward from the Common Councilman, we come ultimately to the "pocket" jimmy—James the less, in more respectful language—which is about 12 inches in length. The Black Museum specimen is of finely tempered steel, and hinged so as to fold in half, in which condition a curate might carry it in his breast pocket without exciting suspicion. The larger sizes divide into two or three lengths, which are screwed together when required for actual use. Some are solid, some of tubular steel, the latter construction giving increased lightness without any sacrifice of strength. Each end terminates in a chisel point, the one straight, the other slightly bent.—*Chambers' Journal.*

